# Comparative transcriptome profiling of multi-ovary wheat under heterogeneous cytoplasm suppression

**DOI:** 10.1038/s41598-019-43277-5

**Published:** 2019-06-05

**Authors:** Jialin Guo, Gaisheng Zhang, Yulong Song, Shoucai Ma, Na Niu, Junwei Wang

**Affiliations:** 1 College of Agronomy, Northwest A & F University, Yangling, Shaanxi 712100 P.R. China; 2 National Yangling Agricultural Biotechnology & Breeding Center, Yangling, Shaanxi 712100 P.R. China; 3 Yangling Branch of State Wheat Improvement Centre, Yangling, Shaanxi 712100 P.R. China; 4 Wheat Breeding Engineering Research Center, Ministry of Education, Yangling, Shaanxi 712100 P.R. China; 5 Key Laboratory of Crop Heterosis of Shaanxi Province, Yangling, Shaanxi 712100 P.R. China

**Keywords:** Plant development, Plant genetics

## Abstract

DUOII is a multi-ovary wheat line with two or three pistils and three stamens in each floret. The multi-ovary trait of DUOII is controlled by a dominant gene, whose expression can be suppressed by the heterogeneous cytoplasm of TeZhiI (TZI), a line with the nucleus of common wheat and the cytoplasm of *Aegilops*. DUOII (♀) × TZI (♂) shows multi-ovary trait, while TZI (♀) × DUOII (♂) shows mono-ovary. Observing the developmental process, we found that the critical stage of additional pistil primordium development was when the young spikes were 2–6 mm long. To elucidate the molecular mechanisms that are responsible for the heterogeneous cytoplasmic suppression of the multi-ovary gene, we RNA-sequenced the entire transcriptome of 2–6 mm long young spikes obtained from the reciprocal crosses between DUOII and TZI. A total of 600 differentially expressed genes (DEGs) was identified. Functional annotation of these DEGs showed that the heterogeneous cytoplasmic suppression of additional pistil development mainly involved four pathways, i.e., chloroplast metabolism, DNA replication and repair, hormone signal transduction, and trehalose-6-phosphate in the primordium development stage, which cooperated to modulate the multi-ovary gene expression under heterogeneous cytoplasmic suppression.

## Introduction

The ovary number in each species is generally constant, but it may vary under specific conditions. An increased ovary number has been reported in *Arabidopsis thaliana*^1^, rice^2^, and wheat^3^. Pistillody and multi-ovary are two different patterns of the increased ovary-number trait in wheat. The former is a non-heritable trait that refers to the existence of multiple pistils, due to the conversion of stamens to carpels^4–7^, and results in a reduced number of stamens, delayed blooming, and partial sterility. The latter is a heritable trait that refers to the existence of two or three pistils and three stamens, and results in two to three seeds per floret. The multi-ovary trait was first reported by Chen *et al*.^8^ who discovered and cultivated “trigrain wheat,” which is an excellent line for studying floral developmental mechanisms and increasing wheat yield. Previous studies mainly focused on the developmental process of floral organs^9^, biochemical basis of seed germination^10^, discovery of molecular markers^11^, gene localization and genetic analysis^12^, and mechanisms of multi-ovary development^13,14^.

In our previous study, we found that the multi-ovary trait in DUOII is controlled by a dominant gene, and that the F_1_s derived from the reciprocal crosses between DUOII and the alloplasmic line TeZhiI (TZI) are phenotypically different^15^. Specifically, the cross between DUOII as a female parent and TZI as a male parent produces a multi-ovary F_1_, whereas, the reciprocal cross produces a mono-ovary F_1_. Since the nuclear DNA involved is the same in both crosses, the multi-ovary trait may be determined by a nuclear-cytoplasmic interaction, and the expression of the multi-ovary gene may be suppressed by the heterogeneous cytoplasm of TZI^16^. However, information on the underlying mechanism that controls the heterogeneous cytoplasmic suppression of the multi-ovary gene in common wheat is limited.

The normal development of floral organs depends on the intricate and precise regulation of gene expression. Genome-wide gene expression profiling by high-throughput sequencing is a powerful method to analyze the molecular regulation mechanisms and acquire candidate genes involved in various processes^17,18^. In wheat, RNA sequencing (RNA-seq) has been successfully applied for the transcriptome analysis of glume development^19^, asymmetry in synthetic and natural allotetraploid lines^20^, fertility conversion in male sterile line^21^, and responses to various biotic and abiotic stresses^22,23^.

In this study, we applied RNA-seq transcriptome analysis to identify differential gene expression patterns in the F_1_s derived from the reciprocal crosses between DUOII and TZI, and gain an insight into the underlying molecular mechanisms that control the heterogeneous cytoplasmic suppression of the multi-ovary gene in wheat. To our knowledge, this is the first report on the universal transcript expression patterns involved in the cytoplasmic suppression of wheat floral meristems.

## Results

### Morphological analysis and cytological examination

**Figure 1:**
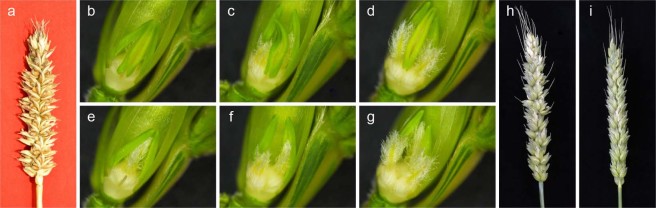
Phenotypic characterization of mono-ovary and multi-ovary florets and spikes of DUOII, Mo_HC, and Mu_NC. (**a**) Spike of DUOII; (**b**,**e**) One ovary in one floret; (**c**,**f**) Two ovaries in one floret; (**d**,**g**) Three ovaries in one floret; (**h**) Spike of Mu_NC; (**i**) Spike of Mo_HC. Mu_NC, F_1_ population derived from a cross between DUOII as female parent and TZI as male parent; Mo_HC, F_1_ population derived from a cross between TZI as female parent and DUOII as male parent.

Generally, there is only one ovary in each floret of wheat. However, DUOII is a multi-ovary wheat variety, which has 2–3 pistils, three stamens per floret, and the genetics of multi-ovary are very stable. Furthermore, it can set 2–3 seeds in one floret (Fig. 1a–g). For the reciprocal crosses between multi-ovary wheat DUOII and alloplasmic cytoplasm wheat TZI, the spike phenotypes of F_1_ plants had similar spike phenotypes (Fig. 1h,i); however, using DUOII as the female parents, the F_1_ plants showed multi-ovary trait with a normal cytoplasm of DUOII (Mu_NC), whereas, using TZI as the female parents, the F_1_ plants showed mono-ovary trait with the heterogeneous cytoplasm of TZI (Mo_HC). The results suggested that the heterogeneous cytoplasm influenced the expression of the multi-ovary trait. Other morphological traits, such as plant height, spike length, spike number, or spikelet number on the main stem spike, were also investigated, but no significant differences were identified between Mu_NC and Mo_HC^16^.

### Identifying the critical stage of additional pistil development

**Figure 2:**
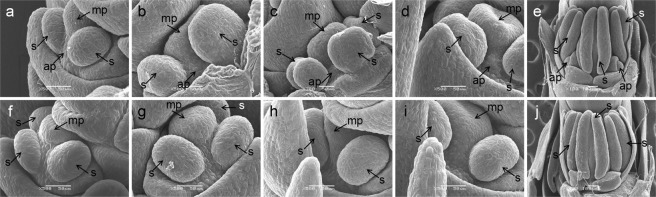
Comparative scanning electron micrograph characterizations of multi-ovary and mono-ovary young spikes. (**a**–**e**) Multi-ovary young spikes; (**f**–**j**) mono-ovary young spikes; (**a**,**f**) 2–3 mm young spikes; (**b**,**g**) 3–4 mm young spikes; (**c**,**h**) 4–5 mm young spikes; (**d**,**i**) 5–6 mm young spikes; (**e**,**j**) about 25 mm spikes. ap: additional pistil; mp: main pistil; s: stamen.

To study the molecular mechanism of the multi-ovary trait, we aimed to identify the critical stage of additional pistil development. In Mu_NC, we observed a small protuberance at the base of the main pistil in the middle floret of 2–3 mm long young spikes, which was between the frontal stamen and lateral stamen; this was considered as the first sign of the additional pistil. The protuberance increased in size with spike development and became obvious in the middle floret of 5–6 mm long young spikes (Fig. 2a–d). However, no protuberance was observed in Mo_HC (Fig. 2f–i). Further, when the spike grew to about 25 mm, the protrusion became bigger, and its position was the same as the final additional pistil (Fig. 2e); and there was still no protuberance in Mo_HC (Fig. 2j). So, the protuberance really was the primordium of additional pistil. Since the developmental process of each floret in a spike was different, the critical stage of additional pistil development was identified as when the young spikes were 2–6 mm long.

### Assessment of RNA-seq Data

**Figure 3:**
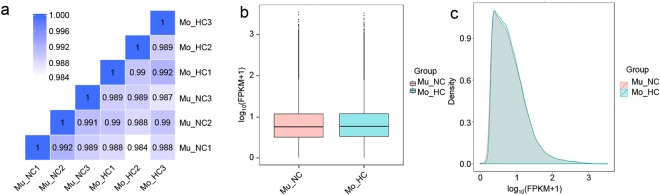
Correlation analysis and distribution of RNA-seq data between Mo_HC and Mu_NC. (**a**) Pearson’s correlation coefficient matrix of RNA-seq between each sample; (**b**) Distribution boxplot graph of fragments per kilobase of transcript per million mapped reads (FPKM) between Mo_HC and Mu_NC. (**c**) Density graph of FPKM for comparing Mu_NC and Mo_HC. Mu_NC, F_1_ population derived from a cross between DUOII as female parent and TZI as male parent; Mo_HC, F_1_ population derived from a cross between TZI as female parent and DUOII as male parent.

RNA-seq produced a total of 94.54 Gb clean data with a GC content of 54.40–55.43%. For each sample, the average clean data were 15.75 Gb, with a Q30 higher than 86.61%, and an alignment efficiency to the reference genome of 70.41–72.47%, of which more than 88% were uniquely mapped reads (Supplementary Table S1). Additionally, Pearson’s correlation between different samples was 0.984–0.992, whereas, R^2^ within the biological replicates was higher than that between Mo_HC and Mu_NC (Fig. 3a). The gene expression levels and sequencing quality were identical in Mo_HC and Mu_NC (Fig. 3b,c). Consequently, the sequencing quality was sufficiently high to allow further analysis.

### Identification of DEGs

**Figure 4:**
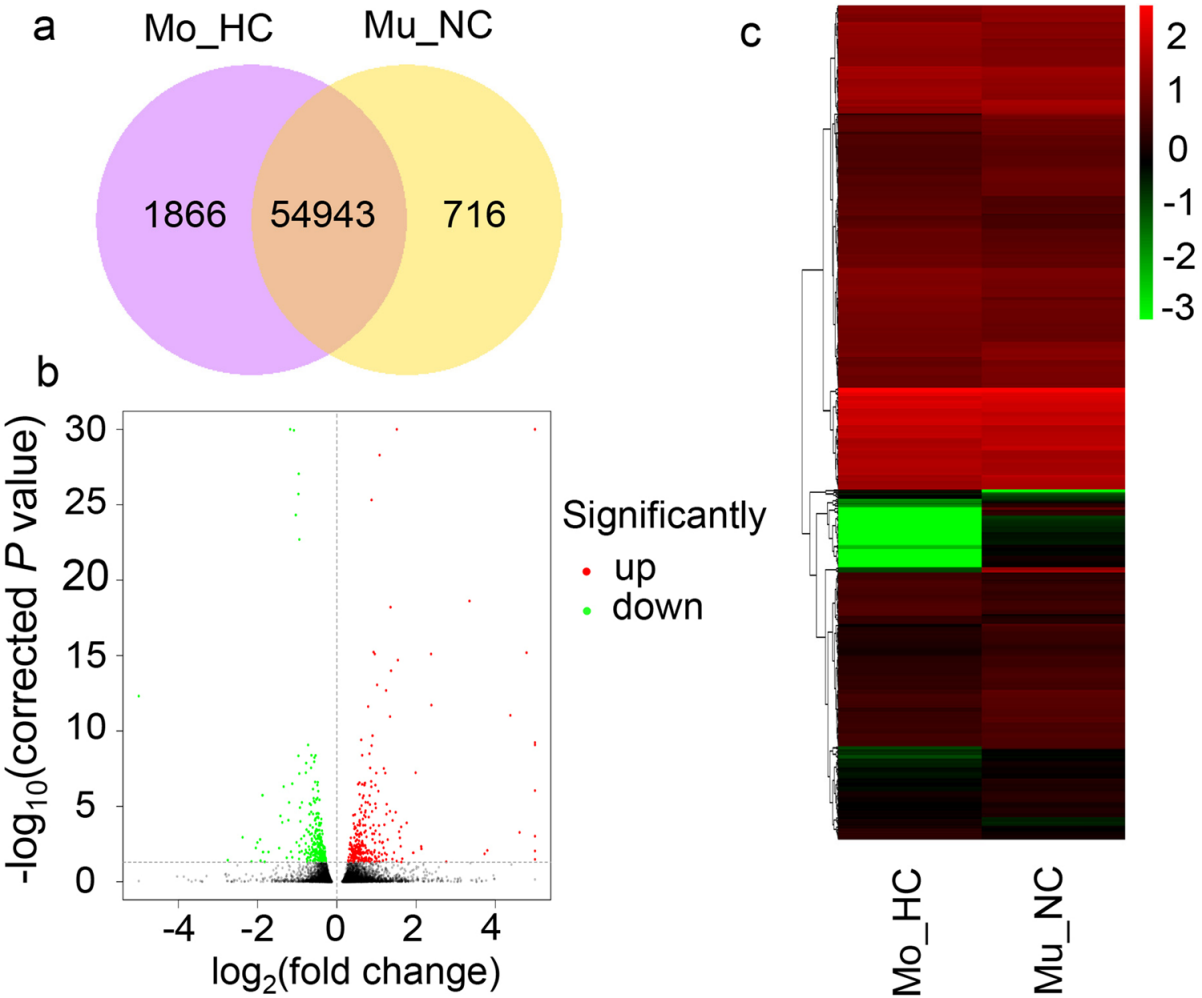
Overlap of expressed genes and identification of differentially expressed genes (DEGs) between Mo_HC and Mu_NC. (**a**) Venn diagram showing the overlap of expressed genes between Mu_NC and Mo_HC. (**b**) Volcano plots showing DEGs between Mu_NC and Mo_HC. (**c**) Hierarchical clustering of DEGs between Mu_NC and Mo_HC. Mu_NC, F_1_ population derived from a cross between DUOII as female parent and TZI as male parent; Mo_HC, F_1_ population derived from a cross between TZI as female parent and DUOII as male parent.

In total, 56,809 and 55,659 genes were co-expressed in Mo_HC and Mu_NC, respectively, and 1,866 and 716 genes were specifically expressed in Mo_HC and Mu_NC, respectively (Fig. 4a). Using an adjusted *P* < 0.05 as a selection criterion, we identified 600 DEGs in Mo_HC; 330 upregulated and 270 downregulated (Fig. 4b; Supplementary Dataset S1). The differential transcription profiles of DEGs were different between Mo_HC and Mu_NC (Fig. 4c), and may possibly be related to the mechanism of heterogeneous cytoplasmic suppression of the multi-ovary gene.

### GO-based functional analysis of DEGs

**Figure 5:**
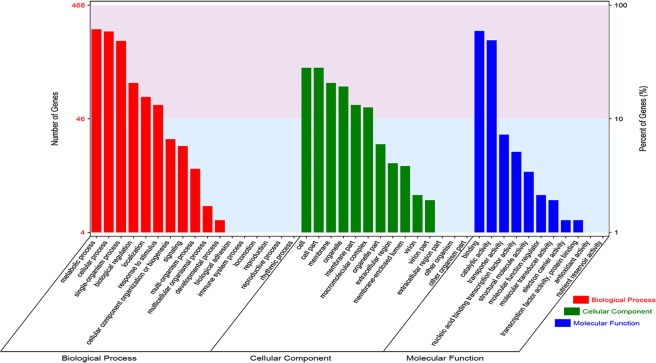
Gene ontology (GO) classification of differentially expressed genes.

Sequence homology analysis showed that 468 DEGs (78% of all DEGs) were associated with at least one GO term and categorized into 42 functional groups at the second level, consisting of 17 groups in biological process, 14 groups in cellular component, and 11 groups in molecular function (Fig. 5). These findings suggested that a wide range of functional genes were related to the process of heterogeneous cytoplasmic suppression of the multi-ovary gene.

To find the most concentrated gene functional groups in DEGs, GO enrichment analysis was performed. Using a corrected *P* < 0.05, 72 GO terms were significantly enriched and included 232 DEGs (Supplementary Dataset S2). Biological process consisted of a major portion of the enriched GO terms (53 terms), followed by molecular function (16 terms) and cellular component (three terms). To explore the relationship of enriched GO terms, we constructed a directed acyclic graph (DAG) using the top 10 enriched GO terms as the master nodes. For biological process, 30 enriched GO terms (56.60% of the total enriched GO terms) were drawn to the DAG divided into three categories: trehalose biosynthetic process (GO:0005992), DNA replication, synthesis of RNA primer (GO:0006269), and spermine biosynthetic process (GO:0006597) (Supplementary Fig. S1a). For cellular component, two enriched GO terms (66.67% of the total enriched GO terms) were drawn to the DAG: checkpoint clamp complex (GO:0030896) and nucleus (GO:0005634). The former was a subset of the latter and also the most detailed GO term in molecular function (Supplementary Fig. S1b). For molecular function, 13 enriched GO terms (81.25% of the total enriched GO terms) were drawn to the DAG divided into four categories: homoserine O-succinyltransferase activity (GO:0008899), sulfate adenylyltransferase (ATP) activity (GO:0004781), adenosylmethionine decarboxylase activity (GO:0004014), and serine-type endopeptidase inhibitor activity (GO:0004867) (Supplementary Fig. S1c).

### KEGG pathway analysis of DEGs

KEGG pathway analysis revealed that 70 terms were enriched for 188 DEGs (Supplementary Dataset S3). Using a corrected *P* < 0.05, we identified eight significantly enriched pathways, consisting of 88 different DEGs, that divided into three subsets according to their location, function, and DEGs in each term (Table 1). Subset I included three terms: DNA replication (20 DEGs), nucleotide excision repair (11 DEGs), and mismatch repair (nine DEGs). The three terms had a containment relationship, located in the nucleus, and cooperated to ensure the accuracy of DNA replication. Except for Novel 12743 (a new gene predicted as replication protein A subunit), the other 19 DEGs were down-regulated, suggesting that the heterogeneous cytoplasm in Mo_HC might negatively affect the quality of DNA replication. Subset II included four terms: photosynthesis-antenna proteins (6), photosynthesis (14 DEGs), carbon fixation in photosynthetic organisms (12 DEGs), and sulfur metabolism (11 DEGs). The four terms were both located in the chloroplast and related to chloroplast metabolism. Except for those in the photosynthesis term, most of the other DEGs were significantly downregulated, suggesting that chloroplast metabolism might be disrupted in Mo_HC and played a role in the suppression of additional pistil development. Subset III included only one term that was plant hormone signal transduction (25 DEGs). Twenty-one DEGs were upregulated, and four DEGs were downregulated. Additionally, of these 25 DEGs, 12 DEGs were identified as transcription factors (TFs), such as auxin response factor (ARF), auxin/indole-3-acetic acid (AUX/IAA), basic region-leucine zipper (bZIP), ethylene-insensitive3-like (EIL), and Orphans, which implied that the expression of many genes related to hormone signal transduction would be influenced. The influenced hormones included auxin, cytokinin, and ethylene, which can influence cell division, growth, and the producing of additional pistils.

**Table 1:** Important Kyoto Encyclopedia of Genes and Genomes (KEGG) pathways identified in Mo_HC compared with that in Mu_NC.

Subsets	KEGG pathways	Location	DEGs	Upregulated DEGs	Downregulated DEGs	Corrected *P*-value
I	DNA replication	Nucleus	20	1	19	3.24E-08
	Mismatch repair	Nucleus	9	1	8	0.011632
	Nucleotide excision repair	Nucleus	11	1	10	0.023256
II	Sulfur metabolism	Chloroplast	11	0	11	0.000516
	Photosynthesis	Chloroplast	14	13	1	0.004396
	Photosynthesis-antenna proteins	Chloroplast	6	0	6	0.006865
	Carbon fixation in photosynthetic organisms	Chloroplast	12	5	7	0.018410
III	Plant hormone signal transduction	Nucleus/Cytosol	25	21	4	0.001473

Note: Mu_NC, F_1_ population derived from a cross between DUOII as female parent and TZI as male parent; Mo_HC, F_1_ population derived from a cross between TZI as female parent and DUOII as male parent; DEG, differentially expressed gene.

### Validation of DEGs through qRT-PCR

To confirm the accuracy and reproducibility of the transcriptome analysis results, 14 candidate DEGs, related to different biological pathways, were randomly selected for qRT-PCR. Linear regression analysis of the fold change of the gene expression rations between qRT-PCR and RNA-seq showed positive correlation (*R*^*2*^ = 0.9521, Supplementary Fig. S2). These results confirmed the reliability of the DEGs obtained by RNA-seq in this study.

## Discussion

DUOII is an excellent variety for studying the mechanisms of the multi-ovary trait and floral development in wheat. The multi-ovary trait of DUOII is controlled by a dominant gene, which is suppressed by the heterogeneous cytoplasm of TZI^16^. So, the suppression of multi-ovary gene is a nuclear-cytoplasm interaction process. Plant genome consists of both nuclear and cytoplasmic genomes, which cooperate to determine plant developmental process. Although the nuclear genome has a predominant role in determining the inheritance of most traits, the cytoplasmic genome also plays an essential role in plant development^24,25^. Chloroplasts are the site of photosynthesis and multiple anabolic reactions essential for growth, development, and reproduction^26^. Signals from chloroplasts can modulate nuclear gene expression and regulate plant development, including flower development^27–29^. Thompson *et al*.^30^ created an *Arabidopsis* mutant lacking the chloroplast-localized rhomboid. The mutant plants that had either a double stigma or a single stigma with distortions in shape and size showed reduced fertility. In the present study, we identified four significantly enriched pathways located in the chloroplast region of Mo_HC, including photosynthesis-antenna proteins, photosynthesis, carbon fixation in photosynthetic organisms, and sulfur metabolism. Of the 43 DEGs involved in these pathways, 18 were upregulated, and the other downregulated. Chloroplast metabolism was disrupted in Mo_HC, which might play an important role in the suppression of additional pistil development.

Floral organs grow from a specialized structure called the shoot apical meristem (SAM), which comprises a pool of stem cells that continuously divide and replenish^31^. SAM produces floral meristems, in which floral organ primordia are formed and developed into organs by coordinated cell division and differentiation^32^. The disruption of nuclear and cell division can cause alterations in cell fate and organ differentiation^33,34^. As shown in Fig. 2, the additional pistil of the multi-ovary wheat was derived from a protuberance between the frontal stamen and lateral stamen. Meanwhile, Wang *et al*.^9^ found that the primordium producing this additional pistil resulted from the abnormal division of the subcortical cell. In the present study, 20 DEGs were related to the DNA replication process, encoding DNA polymerase alpha-primase complex subunit, DNA polymerase epsilon subunit 2, mini-chromosome maintenance (MCM) complex subunits 4, 5, and 6, proliferating cell nuclear antigen (PCNA), and replication protein A (RPA) subunits 1B, 2A, and 2B. All these products play an important role in DNA replication and repair, as well as in cell proliferation; they also cooperate to ensure the accuracy of DNA replication, and coordinate the timing and order of cell cycle events^35,36^. Except for Novel12743, 19 DEGs were down-regulated in Mo_HC, suggesting that DNA replication process plays an essential role in the differentiation of additional pistil primordium, and heterogeneous cytoplasm can disrupt DNA replication and cell cycle events, inhibiting the differentiation of additional pistil primordium.

The initiation of a floral organ is a major step in a plant’s life cycle, and the timing and positioning of floral organ initiation are fundamental aspects of plant inflorescence architecture^37,38^. Plant hormones play a crucial role in determining inflorescence architecture, and disruption of the hormone status alters inflorescence morphology by modulating cell division and differentiation in the inflorescence primordium^39,40^. For instance, when hormone levels and distribution are disrupted in a *vrs2* barley mutant, the two-rowed pattern of spikelets at the base and center of the inflorescence is altered to a six-rowed pattern^41^. In Mo_HC, 25 DEGs were significantly enriched in the plant hormone signal transduction pathway, of which 12 DEGs were TFs. The results showed that the expressions of genes related to hormones, including auxin, cytokinin, and ethylene, were disrupted and could lead to varied hormone signals in Mo_HC.

Disrupted chloroplast metabolism might play an important role in the suppression of additional pistil development. And in the nucleus, the changed DNA replication and hormone signal transduction processes suppressed the differentiation of additional pistil. What act as the messenger to transmit signal between chloroplast and nucleus? Sugars, the main photosynthetic products, not only serve as energy sources in diverse plant functions, but also act as signaling molecules and osmotic regulators, regulating floral signal transduction^42,43^. In the DAG analysis of enriched GO terms in biological process, the DAG divided into three categories: trehalose biosynthetic process, DNA replication, synthesis of RNA primer, and spermine biosynthetic process (Supplementary Fig. S1a). Interestingly, six DEGs of trehalose biosynthetic process were engaged in a special KEGG pathway: starch and sucrose metabolism pathway, which implied that these DEGs might play an important role in the suppression of additional pistil development. Functional analysis showed that the products of these six DEGs were all related to trehalose-6-phosphate (T6P).

T6P is the metabolic precursor of the non-reducing disaccharide trehalose, generated from glucose-6-phosphate (G6P) and UDP-glucose by trehalose-6-phosphate synthase (TPS). Previous studies have shown that T6P transmits signals to the nucleus and chloroplast that subsequently modulate the chloroplast metabolism and nuclear gene expression^44,45^. Additionally, T6P interacts with hormones to influence floral signals^43^. T6P is further metabolized to trehalose by trehalose-6-phosphatase (TPP)^45,46^, and can regulate cell division, differentiation, and plant architecture^47–49^. *RAMOSA3* encodes TPP expressed in discrete domains subtending axillary inflorescence meristems to establish the correct identity and determinacy of axillary meristems in female inflorescences. The *ramosa3* maize mutant plants have ears with additional abnormal branches at their bases generated from abnormal axillary meristems^44^. We also found that the additional pistils in multi-ovary wheat were generated from additional axillary meristems at the base of the native ovary. Interestingly, of the six DEGs involved in trehalose biosynthetic process, five DEGs encoded TPP, and one DEG encoded TPS. Therefore, T6P might play an important role in the suppression of additional pistil development in Mo_HC.

**Figure 6:**
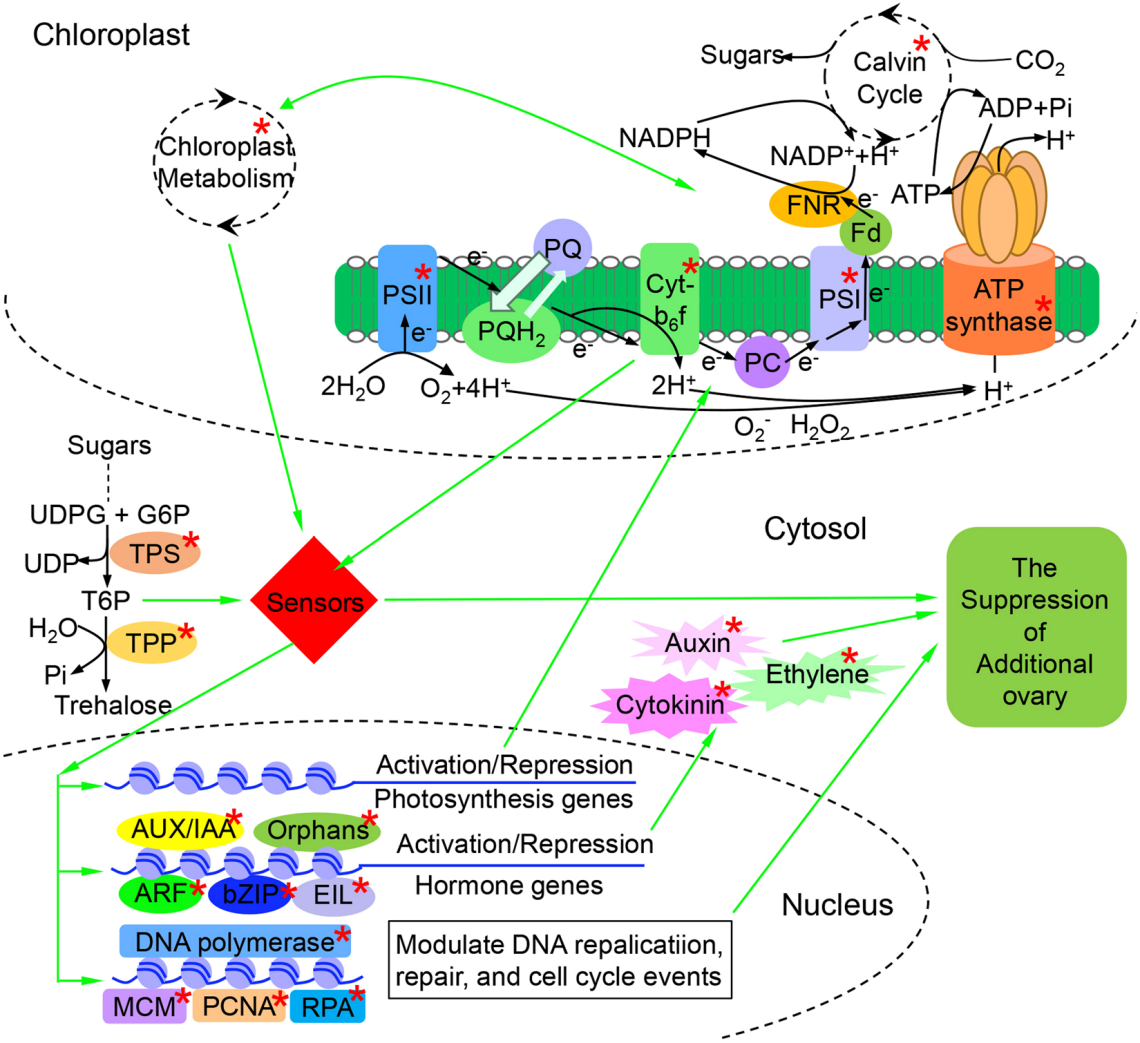
Hypothetical signaling pathway related to heterogeneous cytoplasm-induced suppression of the multi-ovary gene. *Indicates differentially expressed parts in Mo_HC and Mu_NC. Mu_NC, F_1_ population derived from a cross between DUOII as female parent and TZI as male parent; Mo_HC, F_1_ population derived from a cross between TZI as female parent and DUOII as male parent.

Based on the relationships among the chloroplasts, nucleus, hormones, and T6P, we created a hypothetical signaling pathway to better understand their interactions (Fig. 6). In the chloroplast, the disrupted photosynthetic process negatively affected the status of sugars, redox/reactive oxygen species, energy, and metabolites, which interacted with the altered T6P levels to send a signal to the nucleus and TFs through some sensors (e.g., SnRK1^46^ and N-PTM^29^). In the nucleus, DNA replication and repair, as well as cell proliferation, were modulated by DNA polymerase, MCM, PCNA, and RPA, which influenced the genome duplication and cell cycle events. Under the activation or inhibition effect of TFs, the expression of genes related to hormones was either triggered or repressed. Then, the hormones (auxin, cytokinin, and ethylene) modulated cell division and differentiation, which were the basis of the additional pistil meristem development. In addition, the expression of photosynthesis-related genes sent an anterograde signal to the chloroplast, which modulated photosynthesis and the chloroplast metabolism processes. In conclusion, the modified interactions among the chloroplasts, nucleus, hormones, and T6P might be responsible for the heterogeneous cytoplasmic suppression of the multi-ovary trait. To confirm the reliability of this signaling pathway, we randomly selected 14 DEGs related to this pathway and used qRT-PCR to investigated their expression level. The results showed that the expression patterns of these genes revealed by qRT-PCR data were consistent with those derived from RNA-seq (Table 2).

**Table 2:** Validation of the RNA-seq expression profiles of selected DEGs by qRT-PCR.

Transcript ID	Description	Biological pathway	TF family	RNA-seq (FPKM)			qRT-PCR Log_2_(FC)
				Mu_NC	Mo_HC	Log_2_(FC)	
Traes_1AS_D0A2B50BE	DNA replication licensing factor MCM6	DNA replication		299.54	204.85	−0.5482	−0.3001
Traes_2DL_CB0208DE0	PCNA	DNA replication		3280.58	2429.58	−0.4333	−0.4483
Traes_2DL_5B051BFD9	DNA polymerase α complex subunit Pn2	DNA replication		1083.57	797.29	−0.4426	−0.3342
TRAES3BF054800030CFD_g	DNA polymerase ε complex subunit 2	DNA replication		710.64	511.81	−0.4735	−0.4176
Traes_1BS_ADCD5C43B	Ethylene response sensor 2	Hormone signal		131.65	230.16	0.8060	0.7714
Traes_7BL_9ED99FB9E1	EIN3-binding F-box protein 1	Hormone signal		658.87	1391.68	1.0787	0.8722
Traes_2AL_05ECC440C	Auxin response factor	Hormone signal	ARF	1776.36	1432.73	−0.3102	−0.2614
Traes_2AL_A26170C43	Two-component response regulator ORR6	Hormone signal	Orphans	208.49	120.75	−0.7879	−0.9778
Traes_4DL_C083C804E	Ethylene-insensitive 3	Hormone signal	EIL	1245.14	1689.90	0.4406	0.5515
Traes_5DS_76A4D5D4E	Auxin-responsive protein IAA30	Hormone signal	AUX/IAA	1447.51	1843.57	0.3489	0.4205
Traes_7DS_E5A18AAA4	Transcription factor HBP-1b(c1)-like	Hormone signal	bZIP	721.96	552.85	−0.3850	−0.3638
TRAES3BF082400010CFD_g	NADP-dependent malic enzyme 1	Carbon fixation		8468.12	6991.78	−0.2764	−0.2315
Traes_1DL_8809CD0A8	Ferredoxin-nitrite reductase	Sulfur metabolism		2298.88	1552.03	−0.5668	−0.3671
Traes_1DL_48CC7D8E0	Trehalose-6-phosphate synthase	Trehalose metabolism		1006.22	803.97	−0.3237	−0.3142

Note: TF, Transcription factor; FPKM: The expected number of fragments per kilobase of transcript per million mapped reads; Mu_NC, F_1_ population derived from a cross between DUOII as female parent and TZI as male parent; Mo_HC, F_1_ population derived from a cross between TZI as female parent and DUOII as male parent; Log_2_(FC), Log_2_(Fold change) = Log_2_(Mo_HC/Mu_NC).

Multi-ovary wheat has the obvious advantage of increased number of grains per spike, thereby potentially increasing the wheat yield. In addition, heterogeneous cytoplasm can suppress the expression of the multi-ovary trait. To our knowledge, this is the first report of the universal transcript expression patterns involved in the cytoplasmic suppression of wheat floral meristems. This signaling pathway provide insights to understand the regulatory role of retrograde signaling from cytoplasm to nucleus, and will be useful in further mechanistic studies on the underlying mechanism that controls the heterogeneous cytoplasm-induced suppression of the nuclear multi-ovary gene in wheat. Our results provide a foundation for understanding the development of the multi-ovary trait in wheat, and can be implemented in future breeding activities focused on the development of high yield wheat cultivars.

## Methods

### Plant materials

In this study, we used two inbred wheat lines (20 years of artificial selfing with no segregation); DUOII, which is a common multi-ovary line, and TZI, which is an alloplasmic line developed by the Key Laboratory of Crop Heterosis of Shaanxi Province, using the nucleus of ‘Chris’ and the cytoplasm of *Aegilops*. In October 2013, DUOII and TZI were sown in the experimental field of Northwest A & F University, Yangling, China (34°91′N, 106°86′E). In May 2014, reciprocal crosses between DUOII and TZI were performed, and the F_1_ seeds were sown in October 2014. In March 2015, 2–6 mm young spikes were hand-dissected from approximately 100 plants in each F_1_ population, immediately frozen in liquid nitrogen, and stored at −80 °C until RNA extraction.

### Morphological analysis and cytological examination

Photographs of the F_1_ spikes were taken using Nikon D600 digital camera (Nikon, Tokyo, Japan), whereas those of the pistils were taken using a Nikon E995 digital camera (Nikon) mounted on a Motic K400 dissecting microscope (Preiser Scientific, Louisville, KY, USA). For cytological examination, young spikes were processed following the method described by Zhang *et al*.^50^ and observed with a JSM-6360LV scanning electron microscope (JEOL, Tokyo, Japan).

### RNA Extraction, RNA-seq library preparation, and sequencing

Young spikes from three biological replicates (0.2 g each) were separately ground into a fine powder in liquid nitrogen by constant crushing using sterilized and chilled pestle and mortar, and subsequently used for total RNA extraction with TRIzol reagent (Invitrogen Life Technologies, Waltham, MA, USA) following the manufacturer’s instructions. RNA degradation and contamination were monitored by performing 1% agarose gel electrophoresis. RNA concentration was measured using Qubit^®^ 2.0 Fluorometer (Life Technologies, Carlsbad, CA, USA) with Qubit^®^ RNA Assay Kit (Life Technologies). RNA purity was tested using NanoPhotometer^®^ spectrophotometer (Implen, Munich, Germany). RNA integrity was assessed using Bioanalyzer 2100 (Agilent Technologies, Santa Clara, CA, USA) with RNA Nano 6000 Assay Kit. Sequencing libraries were generated using NEBNext^®^ Ultra^TM^ RNA Library Prep Kit for Illumina^®^ (NEB, Ipswich, MA, USA), following the manufacturer’s instructions, and index codes were added to attribute sequences to each sample. Index-coded samples were clustered using cBot Cluster Generation System with TruSeq PE Cluster Kit 3-cBot-HS (Illumina, San Diego, CA, USA), following the manufacturer’s instructions. RNA-seq was performed by Beijing Novogene Technologies (Beijing, China) using Illumina Hiseq platform, and 150 bp paired-end reads were generated.

### Sequence alignment

To obtain high-quality, clean reads, we removed all reads with adaptor sequences, with a percentage of ambiguous bases (N) higher than 10%, and with a percentage of low-quality bases (Q ≤ 20) higher than 50%. The Q30 and GC content of clean data were calculated, and the paired-end clean reads were aligned to the reference genome using TopHat 2.0.12^51^. The reference genome and gene model annotation files were obtained from ftp://ftp.ensemblgenomes.org/pub/release-25/plants/fasta/triticum_aestivum/dna/, and the index of the reference genome was constructed using Bowtie 2.2.3^52^.

### Measurement of gene expression levels and detection of differentially expressed genes (DEGs)

The expected number of fragments per kilobase of transcript per million mapped reads (FPKM) was used for estimating the gene expression levels^51^. The number of reads mapped to each gene was counted by HTSeq. 0.6.1^53^, and then the FPKM of each gene was calculated based on the length of the gene and the number of mapped reads. Differential expression analysis between Mu_NC and Mo_HC was performed using DESeq. 1.18.0 for R^54^. The resulting *P*-value was adjusted using the Benjamini-Hochberg procedure for controlling the false discovery rate. An adjusted *P*-value was computed by DESeq for each gene and those with an adjusted *P* < 0.05 were assigned as DEGs.

### Functional analysis of DEGs

To perform functional annotation, the identified transcript genes were annotated using the National Center for Biotechnology Information (NCBI) non-redundant protein database (Nr) and the Swiss-Prot database. Gene Ontology (GO) enrichment analysis of DEGs was implemented by GOseq for R^55^, in which the gene length bias was corrected. GO terms with corrected *P* < 0.05 were considered significantly enriched by DEGs. Following this, we employed topGO^56^ to constructed the DAG using the top 10 enriched GO terms as the master nodes. For the Kyoto Encyclopedia of Genes and Genomes (KEGG) analysis, statistical enrichment of DEGs in the KEGG pathways was tested by KOBAS^57^. In addition, transcription factors (TFs) were identified by iTAK^58^.

### Validation of DEG expression with quantitative reverse transcription PCR (qRT-PCR)

To validate the RNA-seq results, qRT-PCR analysis was performed. Primers for qRT-PCR were designed using Primer Primer 5.0 software (Primer, Canada) and were synthesized by Invitrogen Life Technologies (Shanghai, China). All primers used for qRT-PCR were list in Supplementary Table S2. qRT-PCR analysis was performed with 2× RealStar Green Power Mixture (Genstar Biosolutions (Beijing) Co., Ltd, China) on a QuantStudio 3 Real-Time PCR System (Applied Biosystems, USA) under the following thermal cycling parameters: 95 °C for 30 s, followed by 40 cycles at 95 °C for 15 s and 60 °C for 1 min. Each reaction mixture was in a final volume of 20 μL, containing 0.4 μL of diluted cDNA and 0.4 μL of each primer, 10 μL of 2× RealStar Green Power Mixture and 0.4 μL of 50× ROX Reference Dye II. All reactions were performed in triplicate on one plate and repeated three times. Wheat actin gene was used as the endogenous control to normalize Ct values of each reaction, and the relative gene expression levels were calculated using the 2^−ΔΔCt^ method^59^.

## Supplementary information

Supplementary information

Supplementary Dataset S1

Supplementary Dataset S2

Supplementary Dataset S3

## Data Availability

The sequence data generated in this study were deposited in the NCBI Sequence Read Archive (Accession number SRP144469). The datasets analyzed during the current study are available from the corresponding author on reasonable request.

## References

[1] Wang G, Zhang Z, Angenent GC, Fiers M (2011). New aspects of *CLAVATA2*, a versatile gene in the regulation of *Arabidopsis* development. J. Plant Physiol..

[2] Yasui Y, Tanaka W, Sakamoto T, Kurata T, Hirano HY (2017). Genetic enhancer analysis reveals that FLORAL ORGAN NUMBER2 and OsMADS3 co-operatively regulate maintenance and determinacy of the flower meristem in rice. Plant Cell Physiol..

[3] Li XF (2011). Identification and expression analysis of genes related to multi-ovary in wheat (*Triticum aestivum* L.). Seed Sci. & Technol..

[4] Meyer VG (1966). Flower abnormalities. Bot. Rev..

[5] Fisher JE (1972). The transformation of stamens to ovaries and of ovaries to inflorescences in *Triticum aestivum* L. under short-day treatment. Bot. Gaz..

[6] Murai K, Takumi S, Koga H, Ogihara Y (2002). Pistillody, homeotic transformation of stamens into pistil-like structures, caused by nuclear-cytoplasm interaction in wheat. Plant J.

[7] Yamamoto M (2013). Identification of a novel homolog for a calmodulin-binding protein that is upregulated in alloplasmic wheat showing pistillody. Planta.

[8] Chen J, Zhang L, Wu B (1983). A preliminary report on the discovery and breeding of the “trigrain wheat”. Acta Agron. Sin..

[9] Wang Y, Ding H, Chen J (1990). Differentiation of additional pistils in a trigrain wheat. J. Lanzhou Univ. (Nat. Sci.).

[10] Chen W, Liu W, Lei Q, Ding H, Wang L (1999). Comparative study of peroxidase isozyme and proteins of trigrain and common wheat in seedlings. Acta Agron. Sin..

[11] Wang JW, Zhang GS, Liu HW, Song YZ, Niu N (2005). Detection of a RAPD marker linked to dominant multi-ovary gene in wheat (*Triticum aestivum*). J. Agric. Biotech..

[12] Peng ZS, Martinek P, Kosuge K, Kuboyama T, Watanabe N (2008). Genetic mapping of a mutant gene producing three pistils per floret in common wheat. J. Appl. Genet..

[13] Guo JL (2015). Optimization of two-dimensional electrophoresis system for the study of multi-ovary character in wheat. Chin. J. Biochem. Mol. Biol..

[14] Guo J (2018). Changes in DNA methylation are associated with heterogeneous cytoplasm suppression of the multi-ovary gene in wheat (*Triticum aestivum*). Crop Pasture Sci..

[15] Ma SC, Zhang GS, Liu HW, Wang JW, Wang XL (2000). Studies on the application of multi-ovary character to hybrid wheat I. Multi-ovary gene loci and cytoplasm effect. Acta Bot. Boreal.-occident. Sin..

[16] Guo J (2017). Special heterogeneous cytoplasm suppresses the expression of the gene producing multi-ovary in common wheat. Euphytica.

[17] Meng C (2017). Transcriptome profiling reveals the genetic basis of alkalinity tolerance in wheat. BMC Genomics.

[18] Yue J, Zhang X, Liu N (2017). Cadmium permeates through calcium channels and activates transcriptomic complexity in wheat roots in response to cadmium stress. Genes Genom..

[19] Zou H (2015). Transcriptome profiling of wheat glumes in wild emmer, hulled landraces and modern cultivars. BMC Genomics.

[20] Wang X (2016). Transcriptome asymmetry in synthetic and natural allotetraploid wheats, revealed by RNA-sequencing. New Phytol..

[21] Ye J (2017). Identification of candidate genes and biosynthesis pathways related to fertility conversion by wheat KTM3315A transcriptome profiling. Front. Plant Sci..

[22] Yadav IS (2016). Comparative temporal transcriptome profiling of wheat near isogenic line carrying *Lr57* under compatible and incompatible interactions. Front. Plant Sci..

[23] Ma J (2017). Transcriptomics analyses reveal wheat responses to drought stress during reproductive stages under field conditions. Front. Plant Sci..

[24] Crosatti C (2013). Cytoplasmic genome substitution in wheat affects the nuclear-cytoplasmic cross-talk leading to transcript and metabolic alterations. BMC Genomics.

[25] Soltani A (2016). Novel nuclear-cytoplasmic interaction in wheat (*Triticum aestivum*) induces vigorous plants. Funct. Integr. Genomics.

[26] Häusler RE, Heinrichs L, Schmitz J, Flügge UI (2014). How sugars might coordinate chloroplast and nuclear gene expression during acclimation to high light intensities. Mol. Plant.

[27] Koussevitzky S (2007). Signals from chloroplasts converge to regulate nuclear gene expression. Science.

[28] Feng P (2016). Chloroplast retrograde signal regulates flowering. Proc. Natl. Acad. Sci. USA.

[29] Susila H, Jin S, Ahn JH (2016). Light intensity and floral transition: chloroplast says “time to flower!”. Mol. Plant.

[30] Thompson EP, Smith SGL, Glover BJ (2012). An *Arabidopsis* rhomboid protease has roles in the chloroplast and in flower development. J. Exp. Bot..

[31] Zhang X (2013). Transcription repressor HANABA TARANU controls flower development by integrating the actions of multiple hormones, floral organ specification genes, and GATA3 family genes in *Arabidopsis*. Plant Cell.

[32] Tabata R (2010). *Arabidopsis* AUXIN RESPONSE FACTOR6 and 8 regulate jasmonic acid biosynthesis and floral organ development via repression of class 1 *KNOX* genes. Plant Cell Physiol..

[33] Costa LM, Gutierrez-Marcos JF, Brutnell TP, Greenland AJ, Dickinson HG (2003). The *globby1-1 (glo1-1)* mutation disrupts nuclear and cell division in the developing maize seed causing alterations in endosperm cell fate and tissue differentiation. Development.

[34] Sun B, Xu Y, Ng K-H, Ito T (2009). A timing mechanism for stem cell maintenance and differentiation in the *Arabidopsis* floral meristem. Genes Dev..

[35] Navas TA, Zhou Z, Elledge SJ (1995). DNA polymerase ε links the DNA replication machinery to the S phase checkpoint. Cell.

[36] MacNeill, S. The eukaryotic replisome: a guide to protein structure and function. (Springer, 2012).

[37] Achard P (2007). The plant stress hormone ethylene controls floral transition via DELLA-dependent regulation of floral meristem-identity genes. Proc. Natl. Acad. Sci. USA.

[38] Chandler JW, Werr W (2014). *Arabidopsis* floral phytomer development: auxin response relative to biphasic modes of organ initiation. J. Exp. Bot..

[39] Liu N (2014). Down-regulation of *AUXIN RESPONSE FACTORS 6* and *8* by microRNA 167 leads to floral development defects and female sterility in tomato. J. Exp. Bot..

[40] Šiukšta R (2015). Inherited phenotype instability of inflorescence and floral organ development in homeotic barley double mutants and its specific modification by auxin inhibitors and 2,4-D. Ann. Bot..

[41] Boden SA (2017). How hormones regulate floral architecture in barley. Nat. Genet..

[42] Van Dijken AJH, Schluepmann H, Smeekens SCM (2004). *Arabidopsis* trehalose-6-phosphate synthase 1 is essential for normal vegetative growth and transition to flowering. Plant Physiol..

[43] Matsoukas IG (2014). Interplay between sugar and hormone signaling pathways modulate floral signal transduction. Front. Genet..

[44] Satoh-Nagasawa N, Nagasawa N, Malcomber S, Sakai H, Jackson D (2006). A trehalose metabolic enzyme controls inflorescence architecture in maize. Nature.

[45] Ponnu J, Wahl V, Schmid M (2011). Trehalose-6-phosphate: connecting plant metabolism and development. Front. Plant Sci..

[46] O’Hara LE, Paul MJ, Wingler A (2013). How do sugars regulate plant growth and development? New insight into the role of trehalose-6-phosphate. Mol. Plant.

[47] Gómez LD, Baud S, Gilday A, Li Y, Graham LA (2006). Delayed embryo development in the *ARABIDOPSIS TREHALOSE-6-PHOSPHATE SYNTHASE 1* mutant is associated with altered cell wall structure, decreased cell division and starch accumulation. Plant J..

[48] Van Houtte H, López-Galvis L, Vandesteene L, Beeckman T, Van Dijck P (2013). Redundant and non-redundant roles of the trehalose-6-phosphate phosphatases in leaf growth, root hair specification and energy-responses in *Arabidopsis*. Plant Signal. Behav..

[49] Chary SN, Hicks GR, Choi YG, Carter D, Raikhel NV (2008). Trehalose-6-phosphate synthase/phosphatase regulates cell shape and plant architecture in *Arabidopsis*. Plant Physiol..

[50] Zhang ZB (2007). Transcription factor *AtMYB103* is required for anther development by regulating tapetum development, callose dissolution and exine formation in *Arabidopsis*. Plant J..

[51] Trapnell C (2010). Transcript assembly and abundance estimation from RNA-Seq reveals thousands of new transcripts and switching among isoforms. Nat. Biotechnol..

[52] Langmead B, Trapnell C, Pop M, Salzberg SL (2009). Ultrafast and memory-efficient alignment of short DNA sequences to the human genome. Genome Biol..

[53] Anders S, Pyl PT, Huber W (2015). HTSeq–a python framework to work with high-throughput sequencing data. Bioinformatics.

[54] Anders S, Huber W (2010). Differential expression analysis for sequence count data. Genome Biol..

[55] Young MD, Wakefield MJ, Smyth GK, Oshlack A (2010). Gene ontology analysis for RNA-seq: accounting for selection bias. Genome Biol..

[56] Faddeeva A (2015). Collembolan transcriptomes highlight molecular evolution of hexapods and provide clues on the adaptation to terrestrial life. PLoS One.

[57] Mao X, Cai T, Olyarchuk JG, Wei L (2005). Automated genome annotation and pathway identification using the KEGG Orthology (KO) as a controlled vocabulary. Bioinformatics.

[58] Zheng Y (2016). iTAK: a program for genome-wide prediction and classification of plant transcription factors, transcriptional regulators, and protein kinases. Mol. Plant.

[59] Livak KJ, Schmittgen TD (2001). Analysis of relative gene expression data using real-time quantitative PCR and the 2^−ΔΔCt^ method. Methods.

