# Supplementary material for: Comparative transcriptome profiling of multi-ovary wheat under heterogeneous cytoplasm suppression
Source: Sci Rep. 2019 Jun 5;9:8301. doi: 10.1038/s41598-019-43277-5 (PMC6549160; doi:10.1038/s41598-019-43277-5)
Supplement: Supplementary file 1 — Supplementary information [file 41598_2019_43277_MOESM1_ESM.pdf]

Supplementary Information

Comparative transcriptome profiling of multi-ovary wheat under heterogeneous cytoplasm suppression

Jialin Guo[1,2,3,4,5], Gaisheng Zhang[1,2,3,4,5*], Yulong Song[1,2,3,4,5], Shoucai Ma[1,2,3,4,5], Na Niu[1,2,3,4,5,] Junwei Wang[1,2,3,4,5]

[1]College of Agronomy, Northwest A & F University, Yangling, Shaanxi, 712100, P.R. China.

[2]National Yangling Agricultural Biotechnology & Breeding Center, Yangling, Shaanxi, 712100, P.R. China.

[3]Yangling Branch of State Wheat Improvement Centre, Yangling, Shaanxi, 712100, P.R. China.

[4]Wheat Breeding Engineering Research Center, Ministry of Education, Yangling, Shaanxi, 712100, P.R. China.

[5]Key Laboratory of Crop Heterosis of Shaanxi Province, Yangling, Shaanxi, 712100, P.R. China.

*Corresponding author: Gaisheng Zhang

College of Agronomy, Northwest A & F University,

Yangling, Shaanxi, 712100, P.R. China.

E-mail: zhanggsh58@aliyun.com

Tel: +86-29-87092085

Fax: +86-29-87092085

Supplementary Figures and Tables

Supplementary Figure S1. Directed acyclic graphs of enriched GO terms.

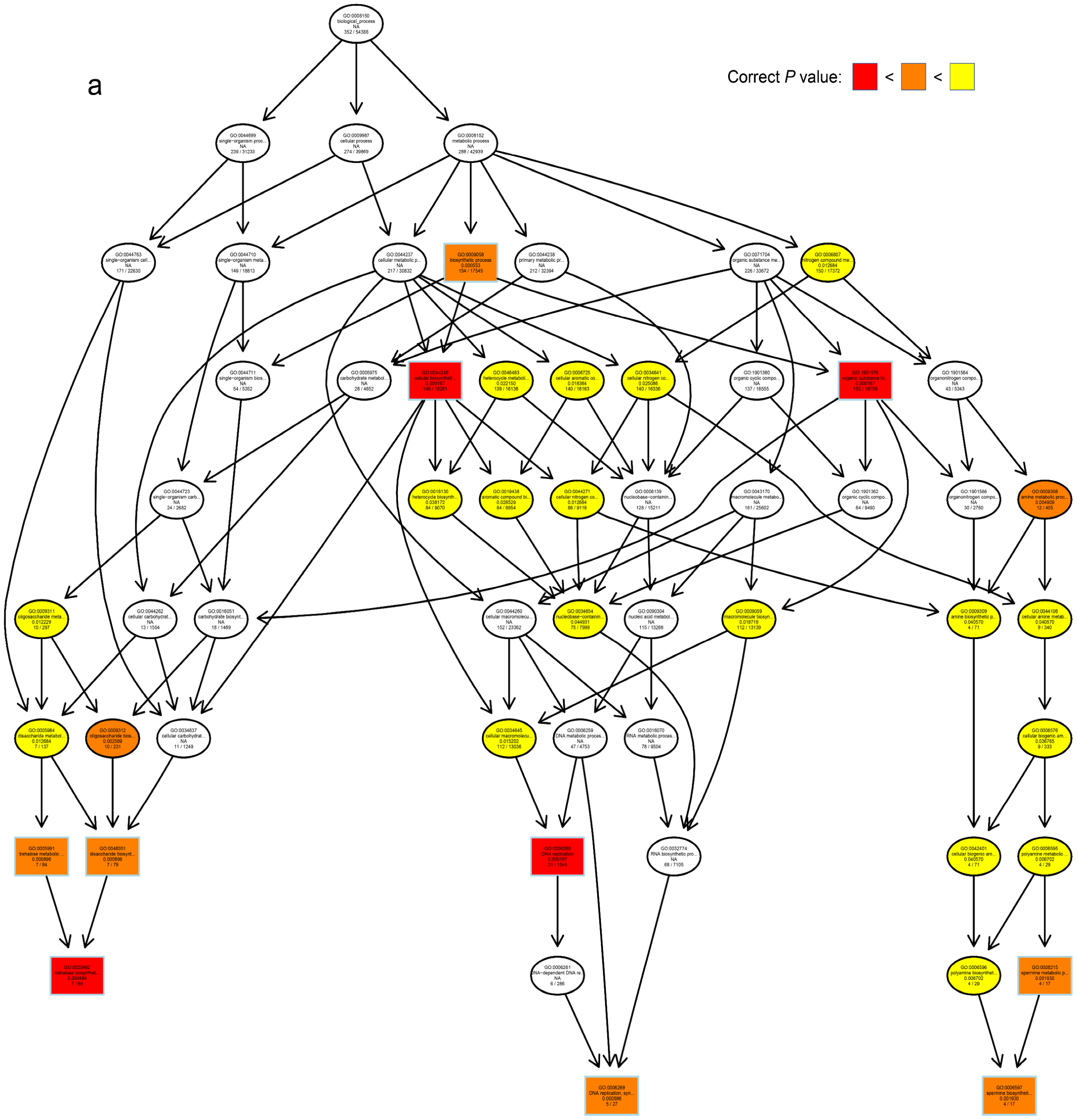

b

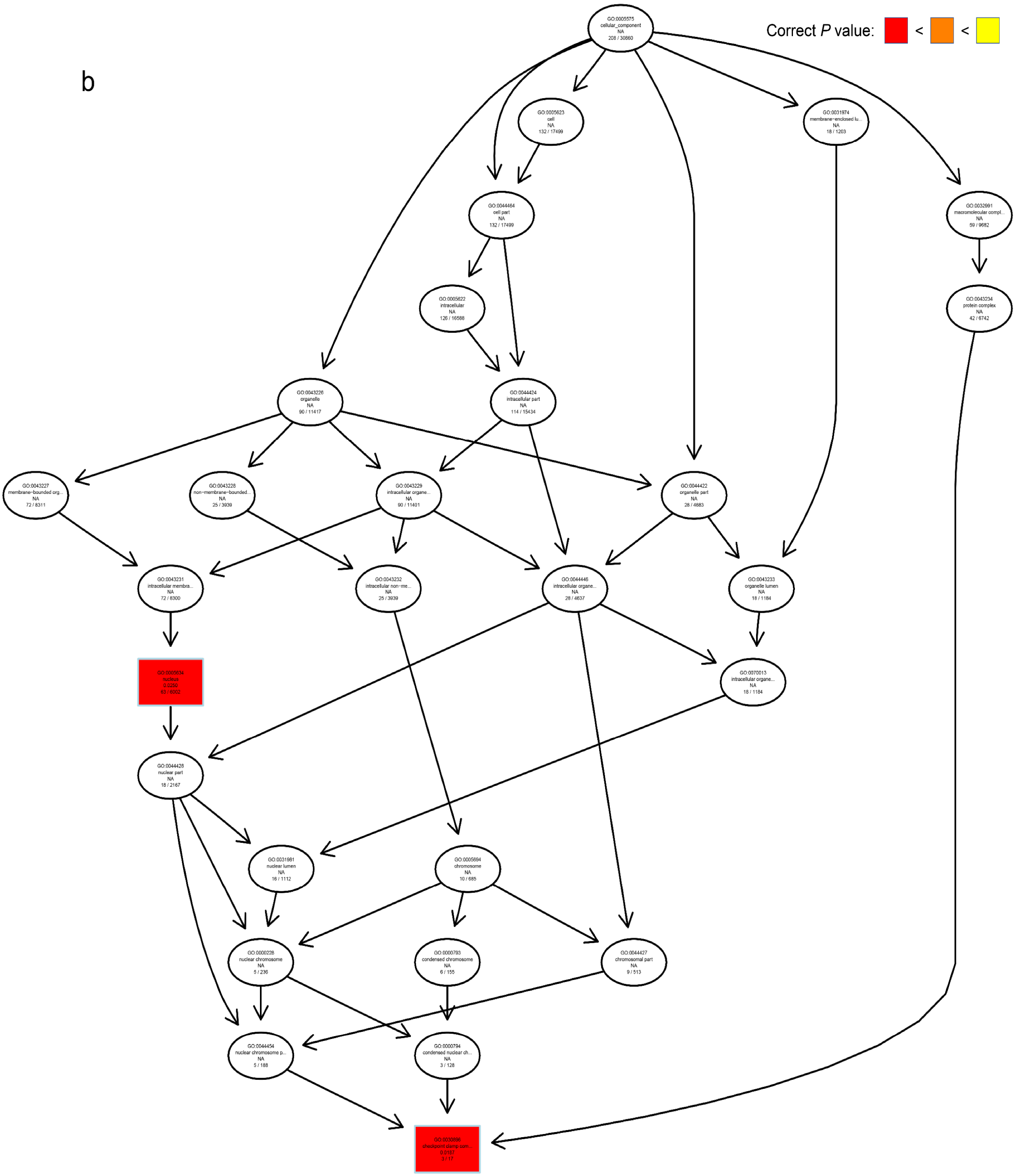

Correct P value: < <
GO:0005575 cellular_component NA 208 / 30860
GO:0005623 cell NA 132 / 17499
GO:0031974 membrane−enclosed lu... NA 18 / 1203
GO:0044464 cell part NA 132 / 17499
GO:0032991 macromolecular compl... NA 59 / 9682
GO:0005622 intracellular NA 126 / 16588
GO:0043234 protein complex NA 42 / 6742
GO:0043226 organelle NA 90 / 11417
GO:0044424 intracellular part NA 114 / 15434
GO:0043227 membrane−bounded org... NA 72 / 8311
GO:0043228 non−membrane−bounded... NA 25 / 3939
GO:0043229 intracellular organe... NA 90 / 11401
GO:0044422 organelle part NA 28 / 4683
GO:0043231 intracellular membra... NA 72 / 8300
GO:0043232 intracellular non−me... NA 25 / 3939
GO:0044446 intracellular organe... NA 28 / 4637
GO:0043233 organelle lumen NA 18 / 1184
GO:0005634 nucleus 0.0250 63 / 6002
GO:0070013 intracellular organe... NA 18 / 1184
GO:0044428 nuclear part NA 18 / 2167
GO:0031981 nuclear lumen NA 16 / 1112
GO:0005694 chromosome NA 10 / 685
GO:0000228 nuclear chromosome NA 5 / 236
GO:0000793 condensed chromosome NA 6 / 155
GO:0044427 chromosomal part NA 9 / 513
GO:0044454 nuclear chromosome p... NA 5 / 188
GO:0000794 condensed nuclear ch... NA 3 / 128
GO:0030896 checkpoint clamp com... 0.0187 3 / 17

c

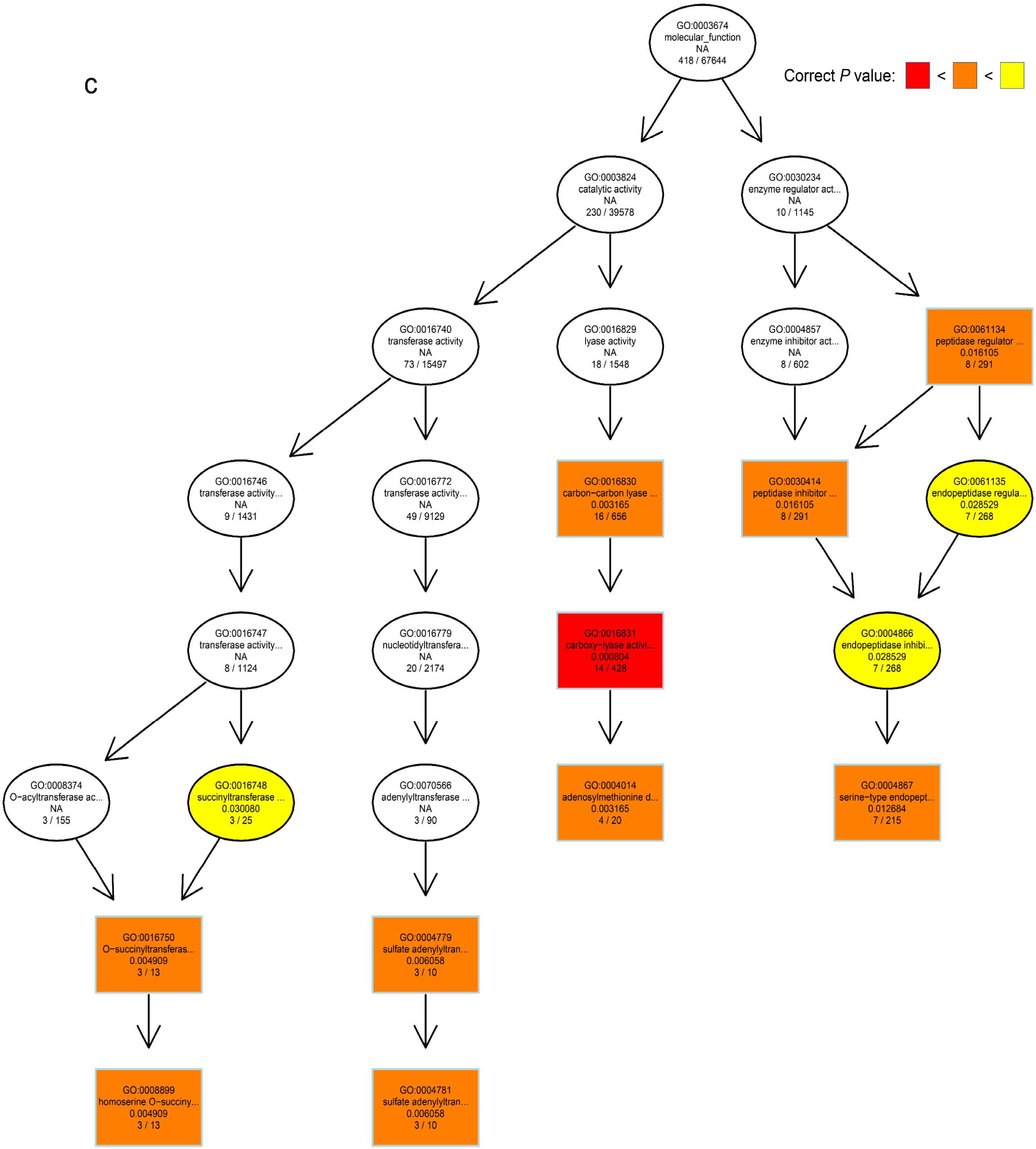

Supplementary Figure S1. Directed acyclic graphs of enriched GO terms. a, b and c represent directed acyclic graphs of enriched GO terms in biological process, cellular component and molecular function, respectively. The colored terms are significantly enriched GO terms. The darker the color, the smaller the corrected *P* value.

Supplementary Figure S2. Comparison of the log_2 fold change of 14 selected DEGs using RNA-seq and qRT-PCR.

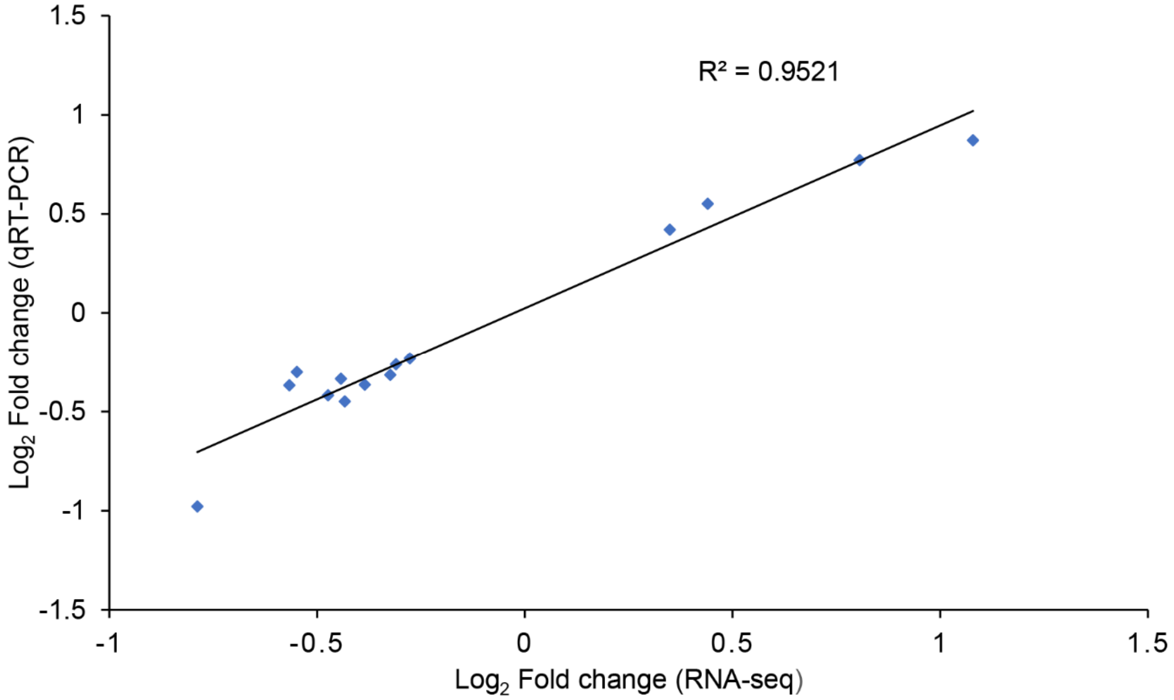

Supplementary Figure S2. Comparison of the log_2 fold change of 14 selected DEGs using RNA-seq and qRT-PCR. Log2 fold change, log2 (Mo_HC/Mu_NC), and Mu_NC, F_1 population derived from a cross between DUOII as female parent and TZI as male parent; Mo_HC, F_1 population derived from a cross between TZI as female parent and DUOII as male parent.

Supplementary Table S1. Evaluation Statistics of transcriptome sequencing data

	Mu_NC1	Mu_NC2	Mu_NC3	Mo_HC1	Mo_HC2	Mo_HC3
Raw Reads	89155990	121248314	97911120	116826696	114348164	111194976
Clean Reads	86600650	117629072	94171412	113291774	110660532	107919556
Clean Bases	12.99G	17.64G	14.13G	16.99G	16.60G	16.19G
Q30 (%)	86.61	89.63	89.88	89.91	89.37	89.24
GC (%)	54.86	54.96	55.27	55.06	55.43	54.4
Total Mapped Reads	61145956 (70.61%)	84826134 (72.11%)	66304722 (70.41%)	80667958 (71.20%)	78182672 (70.65%)	78207164 (72.47%)
Multiple Mapped Reads	6867312 (11.23%)	9564989 (11.28%)	7682207 (11.59%)	9301017 (11.53%)	8792795 (11.25%)	8626730 (11.03%)
Uniquely Mapped Reads	54278644 (88.77%)	75261145 (88.72%)	58622515 (88.41%)	71366941 (88.47%)	69389877 (88.75%)	69580434 (88.97%)

Note: Mu_NC, F_1 population derived from a cross between DUOII as female parent and TZI as male parent; Mo_HC, F_1 population derived from a cross between TZI as female parent and DUOII as male parent.

Supplementary Table S2. Primers used for qRT-PCR

Primer name	Sequence (5' to 3')	Annotation
Actin-Forward	CTCCCTCACAACAACCGC	Actin
Actin-Reverse	TACCAGGAACTTCCATACCAAC	
Traes_1AS_D0A2B50BE-Forward	AGAGGTTTGTGATGGAGCAC	MCM6-like
Traes_1AS_D0A2B50BE-Reverse	TCTCAACCTTTTCAGCATCGG	
Traes_1BS_ADCD5C43B-Forward	TCCCATACAATCCATAACAGCG	Ethylene receptor
Traes_1BS_ADCD5C43B-Reverse	CTGCTTGTGTCTGCCTTTATTG	
Traes_1DL_48CC7D8E0-Forward	GGGCTGAAACTTTTGTATGTGAG	Trehalose-6-phosphate synthase
Traes_1DL_48CC7D8E0-Reverse	GAAACCCAATATGAGCAAACGG	
Traes_1DL_8809CD0A8-Forward	AAACTTCCCTGATTCCCCTG	Ferredoxin--nitrite reductase
Traes_1DL_8809CD0A8-Reverse	CTCCCCTGCACTATCTGAAAC	
Traes_2AL_05ECC440C-Forward	GTCTCAAGTTCTGTCTCTGCC	Auxin response factor
Traes_2AL_05ECC440C-Reverse	ACCTTTGTATATGTCCGCACC	
Traes_2AL_A26170C43-Forward	TGTGGCTGGATTAAACTGGG	OsRR6 type-A response regulator
Traes_2AL_A26170C43-Reverse	TGACATTCCACGAGACAAGAC	
Traes_2DL_5B051BFD9-Forward	AGATGGAGAAACTGGTAAGCG	DNA polymerase alpha complex subunit
Traes_2DL_5B051BFD9-Reverse	GCAGTATACAAGACGGAGCAC	
Traes_2DL_CB0208DE0-Forward	TGTCACTTACCTTTGCTCTGAG	PCNA
Traes_2DL_CB0208DE0-Reverse	AGCCCATCTCACCAATCTTG	
Traes_4DL_C083C804E-Forward	TCCCTTCCTAGCTCTCCAAG	Ethylene-insensitive 3
Traes_4DL_C083C804E-Reverse	CCACTCAAGCAACTCTCCC	
Traes_5DS_76A4D5D4E-Forward	CATTGCAGCCTCTCAAAACAG	Auxin-responsive Aux/IAA factor
Traes_5DS_76A4D5D4E-Reverse	GTTTGCTTCGCTCCTTTCTG	
Traes_7BL_9ED99FB9E1-Forward	CGTGTCTGGTCTCATCTGTTC	EIN3-binding F-box protein 1-like
Traes_7BL_9ED99FB9E1-Reverse	GAGTGAGGGTGAGGTTTGTG	
Traes_7DS_E5A18AAA4-Forward	AAGTCCGACGTGTTCCAC	Transcription factor HBP-1b(c1)-like
Traes_7DS_E5A18AAA4-Reverse	ATATCAAGGTCGCTCAAGGTG	
TRAES3BF054800030CFD_g-Forward	CCGGCAAGATCTCCTTTACAG	DNA polymerase epsilon complex subunit
TRAES3BF054800030CFD_g-Reverse	AGAGGCAATGGACACAGATG	
TRAES3BF082400010CFD_g-Forward	ATCCATCTTTCCACCCTTCAC	NADP-dependent malic enzyme 1
TRAES3BF082400010CFD_g-Reverse	GCTCTCCGCATATTTCTCCAG	
